# Natural Products as a Source for New Leads in Cancer Research and Treatment

**DOI:** 10.1155/2018/8243680

**Published:** 2018-07-15

**Authors:** Célia Cabral, Thomas Efferth, Isabel M. Pires, Patricia Severino, Marco F. L. Lemos

**Affiliations:** ^1^Centre of 20th Century Interdisciplinary Studies (CEIS 20), University of Coimbra, Coimbra, Portugal; ^2^Coimbra Institute for Clinical and Biomedical Research (iCBR), Faculty of Medicine, University of Coimbra, Coimbra, Portugal; ^3^CNC.IBILI Consortium & CIBB Consortium, University of Coimbra, 3000-548 Coimbra, Portugal; ^4^Department of Pharmaceutical Biology, Institute of Pharmacy and Biochemistry, Johannes Gutenberg University, Mainz, Germany; ^5^Hypoxia and Tumour Microenvironment Group, School of Life Sciences, Faculty of Health Sciences, University of Hull, UK; ^6^Laboratório de Nanotecnologia e Nanomedicina (LNMED), Instituto de Tecnologia e Pesquisa (ITP), Universidade Tiradentes (UNIT), Farolândia, Aracaju, SE, Brazil; ^7^MARE-Marine and Environmental Sciences Centre, ESTM, Instituto Politécnico de Leiria, Peniche, Portugal

Cancer is a general term that refers to over 100 distinct pathologies affecting many different tissues and cell types. However, all forms of cancer are characterised by abnormal cell growth resulting from inherited or environmentally induced genomic instability and mutations. Cancer involves cellular transformation, dysregulation of apoptosis and other types of cell death, increased proliferation, angiogenesis, immune evasion, inflammatory responses, and, ultimately, metastatic spread.

The development of a new class of anticancer drugs that lacks the toxicity of conventional chemotherapeutic agents unaffected by common mechanisms of chemoresistance would be a major advance in cancer therapeutics. Several important chemotherapy agents, such as taxanes, certain topoisomerase inhibitor, and* Vinca* alkaloids, were originally identified from natural sources. Therefore, with a myriad of organisms yet to be explored, and new technologies employed, biologically active compounds obtained from natural products will certainly continue to offer vast opportunities as sources of new anticancer therapeutic leads.

For this special issue, focusing on natural products as source for new leads in cancer research and treatment, we have invited authors to contribute with research and review articles illustrating and stimulating the continuing effort in drug discovery and drug development using natural products as source of bioactive molecules in cancer research and treatment. Studies relevant to unveiling of potential targets in cancer biology and the development of innovative therapeutic strategies were also welcome.

From submissions, nine high-quality manuscripts were selected covering the above-mentioned topics.

With the aim of providing a good coverage of the state of the art of this field, this special issue starts with three review papers, followed by six research papers. The first paper (by P. A. Ayeka) is a critical review on mushroom immune modulating potential in cancer. This is followed by another review regarding anticancer properties of natural products, especially essential oils (by K. Blowman et al.). The review third paper dealt with the main characteristics of the studies using flavonoids in the treatment of hepatocellular carcinoma in murine models (by E. R. García et al.).

The remaining papers described original research studies focused on a wide range of topics regarding novel anticancer actions of natural products from different sources, J.-T. Pai et al. revealed that suppression lung cancer cells migration and invasion with propolin C treatment occurred through EMT regulation. The authors also showed how downregulation EGFR-mediated PI3K/Akt and ERK signaling pathways involved in propolin C-regulated EMT in EGFR-mutated lung cancer cells. L. Yanwei et al. proposed that the traditional herbal formula NPC01 could exert its antitumor effect by suppressing the PI3K/Akt/mTOR signaling pathway. The study performed by M. Valenzuela et al. showed novel research concerning anticancer properties of selected grape juice extracts (GJE) by revealing selective cytotoxicity and the ability to reduce invasiveness of colon cancer cells. H. Li et al. evaluated tumor growth inhibition of human hepatocellular carcinoma HepG2 cells in a nude mouse xenograft model by the total flavonoids from* Arachniodes exilis*. Next, the MMP inhibitory effects of flavonoid glycosides from edible medicinal halophyte* Limonium tetragonum* were evaluated in a study by Bae and colleagues. Finally, the last study in this special issue, conducted by Y. Zhu and S. Bu, evaluated the effect of curcumin in human pancreatic cancer cells, revealing that it induces autophagy, apoptosis, and cell cycle arrest.

Overall, the current special issue on natural products as leads for the development of novel anticancer drugs clearly demonstrates that natural resources are still very attractive to find and develop novel tools for the treatment of cancer patients. Taking the phytochemical isolation of novel compounds and their cellular and molecular investigation in* in vitro* cancer cells as a basis,* in vivo* experimentation and clinical trials in human cancer patients have to follow to pave the way for new drugs with better pharmacological features concerning efficacy to kill tumor cells and safety to spare side effects towards normal tissues.

*Célia Cabral* *Thomas Efferth* *Isabel M. Pires* *Patricia Severino* *Marco F. L. Lemos*

